# SympCoughNet: symptom assisted audio-based COVID-19 detection

**DOI:** 10.3389/fdgth.2025.1551298

**Published:** 2025-03-12

**Authors:** Yuhao Lin, Xiu Weng, Bolun Zheng, Weiwei Zhang, Zhanjun Bu, Yu Zhou

**Affiliations:** ^1^ School of Automation, Hangzhou Dianzi University, Hangzhou, China; ^2^ Pediatrics Department, The Third Affiliated Hospital of Wenzhou Medical University, Wenzhou, China; ^3^ Infectious Disease Department, The Third Affiliated Hospital of Wenzhou Medical University, Wenzhou, China; ^4^ Hangzhou Feiyang Biotechnology Co. Ltd., Hangzhou, China

**Keywords:** computational intelligence, multi-modal learning, COVID-19, audio analysis, medical diagnosis

## Abstract

COVID-19 remains a significant global public health challenge. While nucleic acid tests, antigen tests, and CT imaging provide high accuracy, they face inefficiencies and limited accessibility, making rapid and convenient testing difficult. Recent studies have explored COVID-19 detection using acoustic health signals, such as cough and breathing sounds. However, most existing approaches focus solely on audio classification, often leading to suboptimal accuracy while neglecting valuable prior information, such as clinical symptoms. To address this limitation, we propose SympCoughNet, a deep learning-based COVID-19 audio classification network that integrates cough sounds with clinical symptom data. Our model employs symptom-encoded channel weighting to enhance feature processing, making it more attentive to symptom information. We also conducted an ablation study to assess the impact of symptom integration by removing the symptom-attention mechanism and instead using symptoms as classification labels within a CNN-based architecture. We trained and evaluated SympCoughNet on the UK COVID-19 Vocal Audio Dataset. Our model demonstrated significant performance improvements over traditional audio-only approaches, achieving 89.30% accuracy, 94.74% AUROC, and 91.62% PR on the test set. The results confirm that incorporating symptom data enhances COVID-19 detection performance. Additionally, we found that incorrect symptom inputs could influence predictions. Our ablation study validated that even when symptoms are treated as classification labels, the network can still effectively leverage cough audio to infer symptom-related information.

## 1 Introduction

Sound has long been a vital health indicator, with clinicians using noises like “whooping” for pertussis or heart sounds via stethoscopes to detect cardiovascular issues. Non-semantic acoustic signals, such as coughs and breathing patterns, have been linked to conditions like stroke, Parkinson’s, and Alzheimer’s (1, 2). These signals, now easily collected via mobile devices, enhance healthcare screening capabilities (3). Audio-based COVID-19 detection offers a low-cost, non-invasive alternative to PCR and CT scans, reducing disruption to daily life while achieving promising results using datasets like Coswara (4), COUGHVID (5), and Covid-19 Sounds (6). However, many datasets are crowdsourced, with self-reported data and severe class imbalances, complicating model training. The UK COVID-19 Vocal Audio Dataset includes respiratory data from 72,999 participants, with 25,766 positive PCR cases, covering coughs, sequential coughs, and breathing sounds alongside self-reported symptoms. Efforts were made to minimize confounding factors, enabling models to learn acoustic features causally related to COVID-19 rather than incidental noise or unrelated variables.

Deep learning has made remarkable strides, demonstrating immense potential in areas such as computer vision (7, 8), natural language processing, and disease diagnosis (9–11). Researchers have extended these advancements to classify audio signals from COVID-19 patients, analyzing health-related acoustic signals like cough and breathing sounds to promote rapid, contactless COVID-19 detection. For instance, Coppock et al. (12) employed the SSAST model, pre-trained on large audio datasets, as a feature extractor, achieving promising results. Similarly, Han et al. (6) pre-processed audio into 0.96 s non-overlapping segments, applied short-time Fourier transform (STFT) and Mel filter banks, and generated log-Mel spectrograms. These spectrograms were fed into the VGGish model to extract fixed-length latent feature vectors through average pooling. While this method innovatively fused features from multiple acoustic modalities, such as cough, breath, and vocal sounds (13), the improvements from integrating these modalities were modest, and the approach did not incorporate symptoms as prior knowledge into the network. In contrast, studies like Canas et al. (14) have emphasized the importance of self-reported symptoms for COVID-19 detection. Han et al. (15) initially explored combining symptom data with audio features for COVID-19 detection using SVM. Our proposed SympCoughNet leverages symptom encoding to apply channel attention weighting to audio features, further investigating different methods of integrating symptom data with audio. This approach utilizes the complementarity of these data types to enhance the accuracy of COVID-19 detection. Experimental results demonstrate that incorporating symptom features provides valuable context, offering a more comprehensive understanding of patient health.

**Figure 1:**
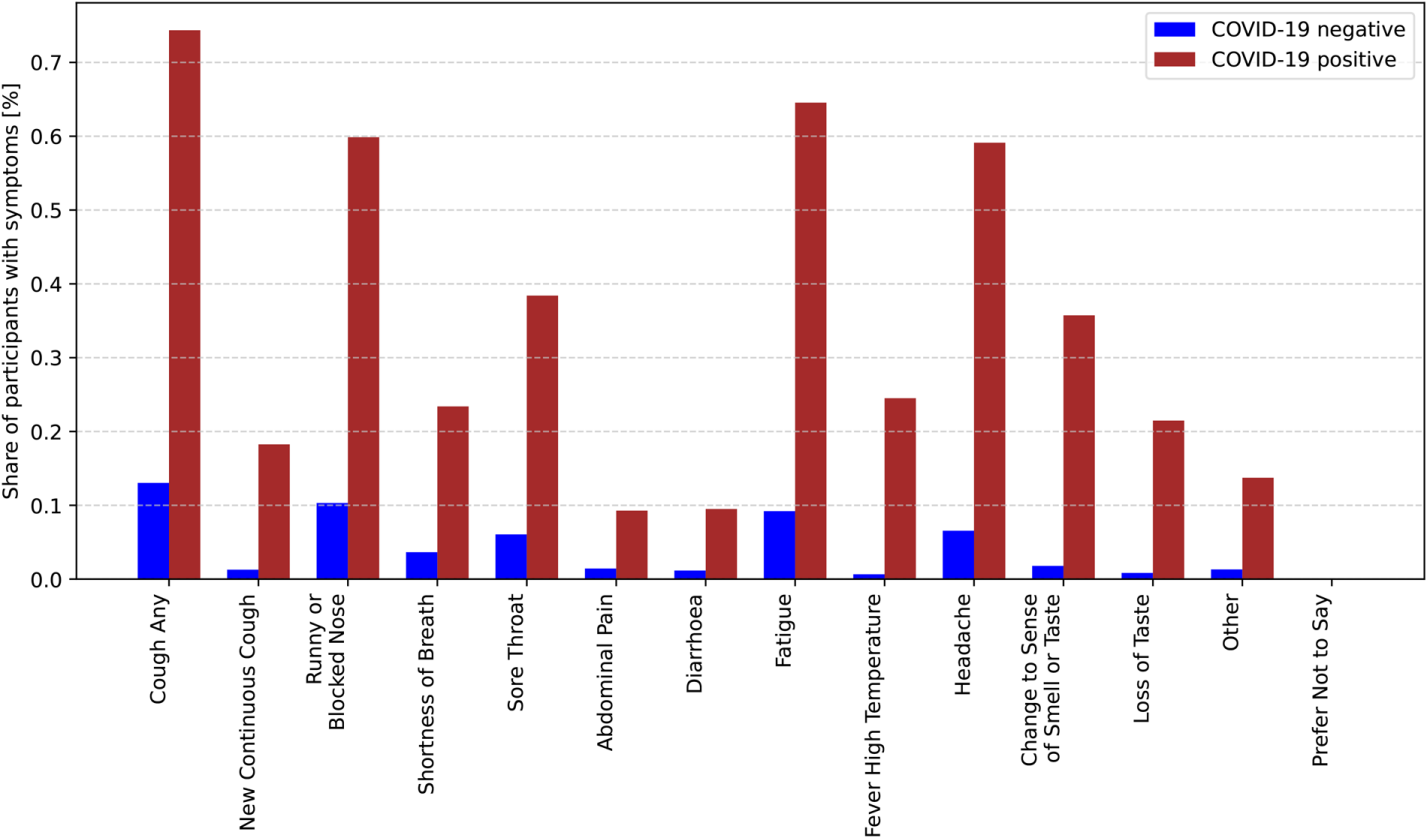
Conditional probabilities of experiencing specific symptoms given a positive COVID-19 diagnosis.

Figure 1 illustrates the distribution of symptoms among COVID-19-positive and COVID-19-negative participants. The *y*-axis represents the percentage of participants exhibiting each symptom, while the *x*-axis lists the symptoms. Among COVID-19-positive participants (red bars), symptoms such as “cough,” “fatigue,” and “headache” have the highest prevalence, with approximately 74% of cases reporting “cough.” Other commonly reported symptoms include “fatigue” (around 65%) and “headache” (over 59%). Additionally, symptoms like “runny or blocked nose” and “changes in sense of smell or taste” are relatively common among positive cases. In contrast, for COVID-19-negative participants (blue bars), the prevalence of all symptoms is significantly lower. The most frequently reported symptom in this group is “cough,” but its occurrence is much lower compared to COVID-19-positive participants. Symptoms such as “abdominal pain,” “diarrhoea,” and “other” are rare in both groups. Notably, the symptom “prefer not to say” appears only in the negative group, and its proportion is extremely low. This chart effectively highlights the differences in symptom distribution between COVID-19-positive and negative cases, underscoring the importance of symptom analysis in COVID-19 diagnosis.

However, the use of symptom-assisted audio methods might lead to classification errors if patients misreport symptoms due to input mistakes or limited symptom awareness. To address this issue, we ablate the symptom attention structure and instead use symptom information as classification labels to train a symptom classifier, aiming to assist patients in filling in symptom information based on cough audio analysis.

We summarized our contributions as follows.

• Our method builds upon an audio-based COVID-19 detection model by computing symptom attention using symptom labels. This symptom attention is then multiplied with each layer’s features to achieve symptom-assisted audio detection.
• To address the issue of patients incorrectly or mistakenly filling in symptom information for various reasons, we use symptom information as classification labels during training to assist patients in providing accurate symptom information.
• In this paper, we explore a novel approach that integrates audio features with symptom information for segment-based COVID-19 detection. Experimental results demonstrate that incorporating symptom priors can significantly enhance the model’s performance.

## 2 Related work

### 2.1 COVID-19 symptoms

We explored how audio features and common COVID-19 symptoms can be effectively integrated into network architectures, emphasizing the critical role of these symptoms in COVID-19 detection. According to the World Health Organization (WHO), fever, cough, and shortness of breath are the primary symptoms of COVID-19. These symptoms can appear either independently or simultaneously. However, these signs are not exclusive to COVID-19 and are also frequently observed in other illnesses, which poses challenges in diagnosis.

Zoabi et al. (16) utilized eight features, including gender, age, history of contact with infected individuals, and other basic clinical characteristics, to predict COVID-19 cases. Their research highlights the significance of leveraging diverse features for accurate diagnosis. Similarly, Fakieh et al. (17) employed statistical analyses, such as ANOVA and t-tests, to evaluate the relationships between demographic factors and symptom variables. They further incorporated machine learning models, utilizing ensemble methods to enhance the accuracy of COVID-19 detection, demonstrating the potential of data-driven approaches. In contrast, Wang et al. (18) reported that some COVID-19 patients did not exhibit typical respiratory symptoms such as cough and fever. Instead, they presented neurological symptoms, including headaches, fatigue, and difficulty walking. This subgroup of patients without respiratory symptoms introduces challenges for audio-based COVID-19 detection, as the absence of characteristic respiratory signs may limit the effectiveness of such methods.

These findings underscore the necessity of adopting a multimodal diagnostic approach. Collecting data from diverse modalities, such as audio features, clinical symptoms, and demographic factors, allows for a comprehensive assessment of the patient’s condition. This integrated evaluation strategy could significantly improve the accuracy and robustness of COVID-19 detection methods, accommodating the variability in symptom presentation and addressing the limitations of single-modality diagnostic techniques.

### 2.2 COVID-19 audio

With the advancement of machine learning, researchers have explored the use of cough as a biomarker for specific diseases (19). Larson et al. (20) stored cough segments from raw audio as single-column vectors, combined them into matrices, and extracted features using PCA. These features were then used to train a random forest classifier, achieving effective cough classification. Liu et al. (21) incorporated Hidden Markov Models (HMMs) to capture temporal information and employed transfer learning for cough classification experiments. Coppock et al. (22) utilized convolutional neural networks to detect COVID-19. Similarly, researchers have leveraged cough audio for the detection of pertussis (23) and tuberculosis (24, 25).

Studies have shown that COVID-19 infection may be associated with pathological changes in the vocal system, a hypothesis based on the fact that voice changes are often linked to vocal system pathologies (26). Asiaee et al. (27) compared sustained vowel /a:/ recordings from Persian speakers who were COVID-19 positive and negative, extracting eight acoustic parameters: F0 and its variation (F0SD), jitter, shimmer, harmonics-to-noise ratio (HNR), the amplitude difference between the first two harmonics (H1-H2), maximum phonation time (MPT), and cepstral peak prominence (CPP). The results showed significant differences in all acoustic parameters except F0 between COVID-19 patients and healthy controls. Moreover, Bartl-Pokorny et al. (26) suggested that COVID-19 infection might be characterized not by a single feature but by a combination of candidate features associated with specific phonation tasks. Madhurananda Pahar et al. (28) evaluated the classification performance of COVID-19 cough data using seven machine learning classifiers, with ResNet50 demonstrating the best performance. Coppock et al. (12) employed Transformer, ResNet, and SVM for COVID-19 identification. Saranga and Kingkor Mahanta et al. (29) applied data augmentation techniques, such as time stretching, pitch scaling, and volume adjustment, to extract MFCC features, which were then fed into a convolutional neural network, achieving promising classification results.

Cough biomarkers have been extensively explored for detecting various diseases, including pertussis and tuberculosis. Recent studies have demonstrated their potential for identifying COVID-19 signals using advanced methods such as deep learning, data augmentation, and acoustic feature analysis. These approaches, including ResNet, Transformers, and MFCC-based techniques, effectively capture disease-specific patterns in cough and voice data, offering robust tools for COVID-19 detection (30–32).

## 3 Method

**Figure 2:**
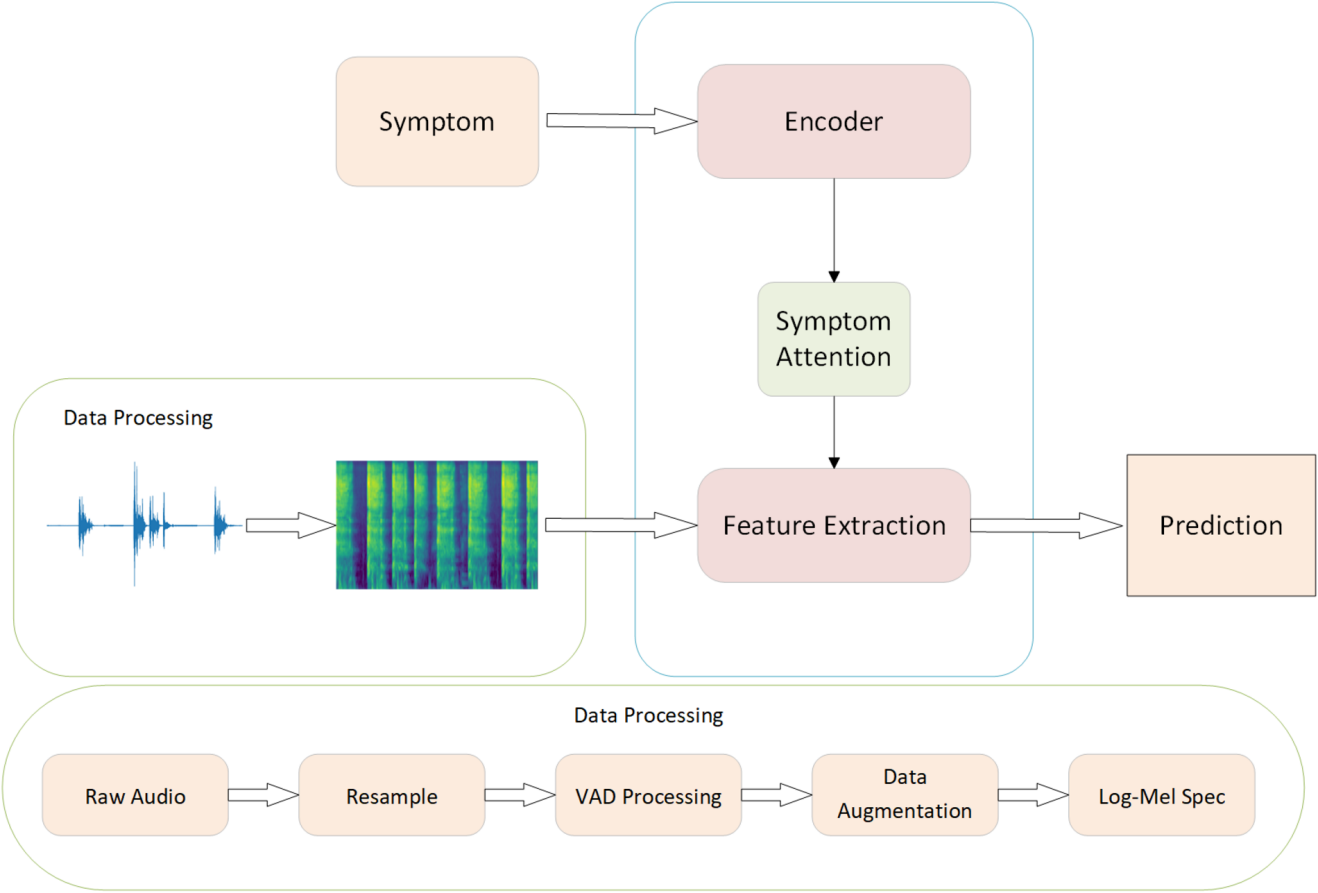
The workflow of SympCoughNet.

The overall Workflow of our symptom-assisted audio detection method is shown in Figure 2. The process begins with data preprocessing of the input raw audio files. Initially, Voice Activity Detection (VAD) and data augmentation techniques are applied to the audio to remove noise segments from the raw recordings. Random noise addition and volume amplification are introduced to enhance the model’s robustness to noise. Similar to previous methods for COVID-19 audio detection, the audio is transformed into log-Mel spectrograms, which are then input into a convolutional neural network for classification.

To handle the varying lengths of audio signals, we standardize the duration of cough signals by looping them to 3.84 s. This duration adequately covers almost all cough lengths and aligns with the average cough duration observed in the dataset. The audio is then framed and windowed with a frame length of 25 ms and a frame shift of 10 ms. The resulting audio segments are filtered using a Hanning window, as shown in Equation 1, to minimize spectral leakage. Next, a Short-Time Fourier Transform (STFT) is applied to obtain the spectrogram. The magnitude spectrum is then filtered using 64 Mel filter banks, and the logarithm is taken to produce log-Mel spectrograms with dimensions of 384 × 64.

(1) $$w(n)=\left\{ \begin{array}{cc} 0.5\left[1-\cos\;\left(\frac{2\pi n}{N-1}\right)\right], & 0\leq n\leq N-1,\\ 0, & \text{otherwise}. \end{array}\right.$$

We utilized 14 symptoms in our analysis: “cough any,” “new continuous cough,” “runny or blocked nose,” “shortness of breath,” “sore throat,” “abdominal pain,” “diarrhoea,” “fatigue,” “fever high temperature,” “headache,” “change to sense of smell or taste,” “loss of taste,” “prefer not to say,” and “other.” These symptoms were encoded as a 14-dimensional one-hot vector, where the presence of a specific symptom was encoded as 1, and its absence was encoded as 0. Based on observations, disease symptoms are closely related to the accuracy of COVID-19 detection. Notably, some patients exhibited no significant symptoms. For these asymptomatic cases, audio features may serve as critical information for effective differentiation.

### 3.1 SympCoughNet

**Figure 3:**
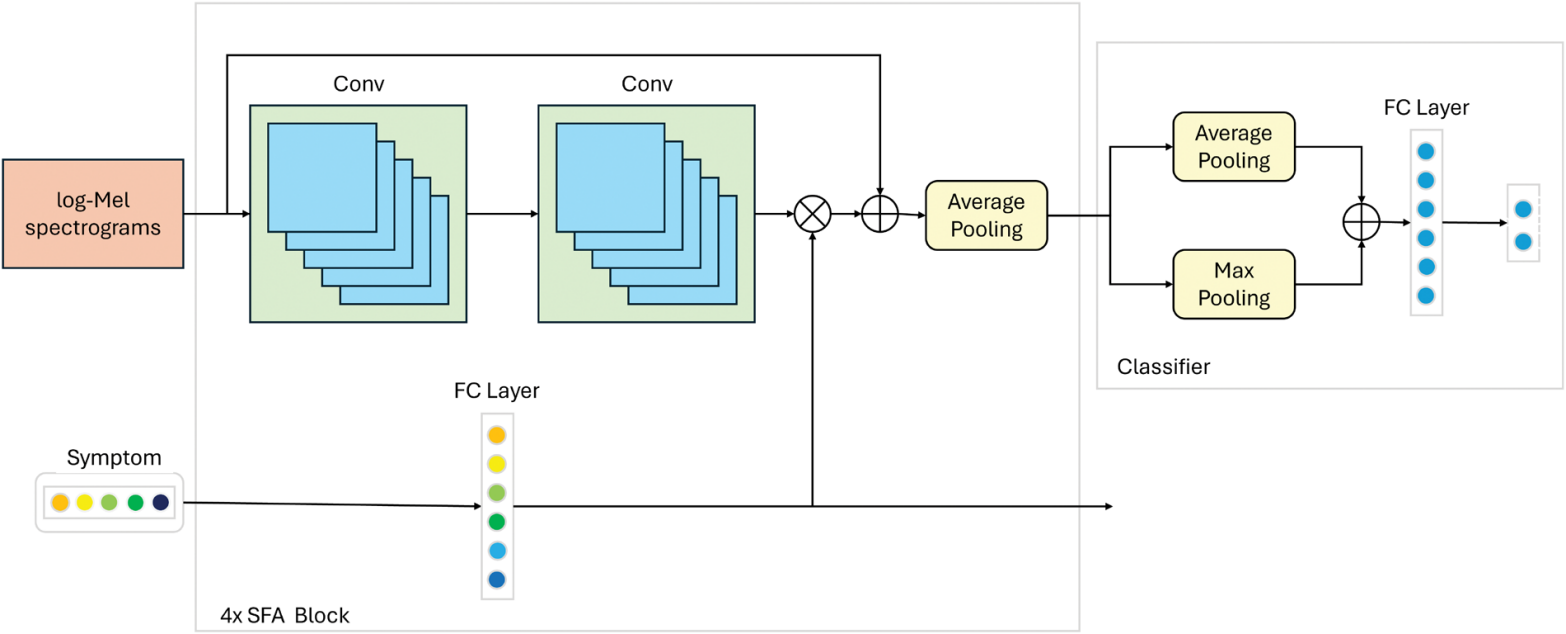
Network structure details of the proposed SympCoughNet. The symbol “×” represents element-wise multiplication, and “+” represents element-wise addition. “4×” indicates that the operation needs to be repeated four times.

To enable rapid and accurate detection of COVID-19 by incorporating symptom priors, we designed a symptom-assisted audio-based neural network, as illustrated in Figure 3. The proposed model architecture consists of three primary modules: Audio Feature Extraction, Symptom Attention, and Classifier. In the Audio Feature Extraction module, we utilize a classic Convolutional Neural Network (CNN) to extract meaningful representations from the input audio data. To improve the efficiency and speed of training, residual connections are integrated into the CNN architecture, which help mitigate gradient vanishing issues and facilitate deeper model training. For encoding symptom information, we employ a Multi-Layer Perceptron (MLP) to process the symptom data into a latent representation. This representation is then used in the Symptom Attention Module, where it is multiplied with the channel features extracted by the CNN. This mechanism dynamically adjusts the importance of different channels based on symptom information, enabling the network to focus on the audio features most relevant to the provided symptoms. By coupling symptom priors with audio features, the network effectively enhances its ability to identify patterns associated with COVID-19. The Classifier module consists of fully connected layers, which aggregate the enhanced features and predict the final classification results. This straightforward yet effective design ensures that the extracted features and attention-weighted information are fully utilized to achieve accurate predictions. Overall, the SympCoughNet leverages both audio and symptom information, providing a robust framework for COVID-19 detection that combines symptom priors with audio signal characteristics.

To integrate symptom prior information with audio data, we designed a Symptom-Fused Attention Block (SFA Block) that combines the two during the feature extraction process. Suppose $M_{\mathrm{in}}^{i}$ and $S_{\mathrm{in}}^{i}$ denote the input audio and symptom information for the i-th SFA Block, respectively. Audio features $f_{c}=Conv(M_{\mathrm{in}}^{i})$ are extracted using two convolutional layers and then reduced to half their original size through average pooling. Meanwhile, symptom information is encoded using a fully connected layer, producing $f_{s}=Fc(S_{\mathrm{in}}^{i})$. The activation function for both the convolutional and fully connected layers is ReLU. The encoded features $f_{s}$ are used to compute the channel attention as $f_{\mathrm{ca}}=Sigmoid(f_{s})$. To avoid information loss, a skip connection is employed. Finally, the output audio and symptom information, $M_{\mathrm{out}}^{i}$ and $S_{\mathrm{out}}^{i}$, for the i-th SFA Block is formulated as follows.

(2) $$M_{\mathrm{out}}^{i}=M_{\mathrm{in}}^{i}+f_{\mathrm{ca}}*f_{c}$$

(3) $$S_{\mathrm{out}}^{i}=Relu(Fc(S_{\mathrm{in}}^{i}))$$

In our Classifier module, we first apply both max pooling and average pooling to the feature maps. The resulting values are then summed together and passed through a fully connected layer, which produces the final prediction results.

### 3.2 Parameter selection

In this section, we provide a detailed explanation of the parameter settings used in our model. Similar to the settings in (6, 12), the input log-Mel spectrograms are shaped as 1×384×64. We repeat the SFA Block four times, with the number of channels progressively set to [64, 128, 256, 512]. Each convolution operation utilizes a kernel size of 3×3, a padding size of 1, and a stride of 1. Correspondingly, the symptom information is encoded into vectors of [64, 128, 256, 512] channels, which are then multiplied element-wise with the results of the convolutional layers. The specific parameter details are summarized in Table 1.

**Table 1:** Network parameter details for symptom-fused attention block (SFA block).

Component	Kernel size
SFA Block 1	3×3×1×64
SFA Block 2	3×3×64×128
SFA Block 3	3×3×128×256
SFA Block 4	3×3×256×512

## 4 Experiments

In this section, we perform a comprehensive evaluation of the performance of our proposed method. First, we provide a detailed explanation of the data preprocessing pipeline.Next, we elaborate on the implementation details of network training, such as the model architecture configuration, hyperparameter selection, training strategies, and optimization methods. Finally, we present the experimental results, including an analysis of performance metrics and ablation studies to verify the contribution of each component to the overall performance.

### 4.1 Data processing

In tasks such as speech recognition and audio classification, the performance of Mel spectrograms has been widely validated as efficient and reliable. Log-Mel spectrograms convert audio time series into a two-dimensional time-frequency representation, significantly reducing the feature dimensions, simplifying computation, and retaining the essential audio information. First, the raw audio file undergoes data preprocessing. Voice Activity Detection (VAD) and data augmentation techniques are applied to process the audio, removing noisy segments from the raw audio. Random noise addition and volume augmentation are used to enhance the model’s robustness to noise. The processed audio is then transformed using Short-Time Fourier Transform (STFT) and Mel filter banks to obtain the log-Mel spectrogram.

We divided the UK COVID-19 Vocal Audio Dataset into training, validation, and testing sets with a ratio of 0.7:0.15:0.15. To prevent potential imbalance between positive and negative samples during the split, we ensured that the proportion of positive to negative samples in each subset closely matched the original dataset’s ratio of 65:35. Data augmentation was applied only to the training set, while the validation and testing sets remained unchanged. The data augmentation process was performed randomly during training, with varying levels of augmentation in each epoch to minimize overfitting.

The UK COVID-19 Vocal Audio Dataset stores audio files in WAV format, which we processed using the librosa library. Librosa automatically normalizes the audio by adjusting the amplitude to the range [−1, 1]. We resampled all raw audio to 48 kHz. To remove the silence and noise segments frequently present in raw audio, we utilized webrtcvad. To maximize data utilization and mitigate overfitting, we applied data augmentation techniques. Volume adjustments were set within a range of −0.05 dB to 0.05 dB, and Gaussian noise with a mean of 0 and variance of 0.05 was added.

Finally, the processed audio was transformed using the Short-Time Fourier Transform (STFT) and passed through a Mel filter bank to obtain the log-Mel spectrograms. The data preprocessing workflow is illustrated in Figure 2.

### 4.2 Experimental setup

To validate the effectiveness of our method, we compare it with several widely used models in the audio domain, including PANN (33), CAM++ (34), EcapaTdnn (35), TDNN (36), Res2Net (37), ResNetSE (38), ERes2Net (39), and HTSAT (40). To ensure a fair comparison, all experiments, except for HTSAT, are conducted with the same data preprocessing techniques.HTSAT is used within a transfer learning framework, with its pre-trained model trained on the ESC-50 dataset. We meticulously followed the original data processing procedures to ensure consistency, resulting in the final experimental outcomes.

During training, we set the batch size to 8 and the initial learning rate to 0.0001. The learning rate is halved every 5 epochs, and a total of 50 epochs are trained. We use the Adam optimizer to optimize the network, with a weight decay of $1\times 10^{-6}$ and a dropout probability of 0.1. The model with the highest accuracy on the validation set after 50 epochs is selected as the final model. Most of the experiments were conducted on an NVIDIA RTX 4090.

### 4.3 Metric

In our experiments, we employed three evaluation metrics, Accuracy, AUROC (Area Under the Receiver Operating Characteristic Curve), and AP (Average Precision)—to comprehensively evaluate the performance of the classification model. Specifically, TP (True Positive) represents cases where both the actual labels and predicted results are positive, FP (False Positive) indicates cases where the actual labels are negative but the predictions are positive, TN (True Negative) represents cases where both the actual labels and predictions are negative, and FN (False Negative) refers to cases where the actual labels are positive but the predictions are negative.

(4) $$\text{Acc}=\frac{\text{TP}+\text{TN}}{\text{TP}+\text{FP}+\text{TN}+\text{FN}}$$

Accuracy measures the proportion of correctly predicted samples out of the total samples, as defined in Equation 4.

AUROC quantifies the classification performance of the model as the area under the ROC curve, which plots the True Positive Rate (TPR) against the False Positive Rate (FPR) across different threshold values.

AP evaluates the model’s overall performance in terms of precision (Precision) and recall (Recall) by calculating the area under the Precision-Recall (PR) curve, capturing its behavior at all threshold levels.

### 4.4 Experimental results

As shown in Table 2, the maximum values for ACCURACY, AUROC, and PR metrics are 100%, while the minimum values are 0%. For all three metrics, higher values indicate better performance. Our method achieved an ACCURACY of 89.30%, an AUROC of 94.74%, and a PR of 91.62%. Compared to the next-best method, HTSAT, our approach improved ACCURACY by 12.96%, AUROC by 11.92%, and PR by 16.28%. These results demonstrate that our method significantly enhances COVID-19 detection performance by incorporating prior clinical symptom information alongside audio data. This further validates the effectiveness of leveraging clinical symptom priors in the diagnosis of COVID-19.

**Table 2:** Performance of different methods in diagnosing COVID-19.

Method	ACC	AUROC	PR
PANN	75.34%	80.92%	72.97%
CAM++	74.80%	79.82%	68.03%
EcapaTdnn	75.40%	81.25%	73.23%
ERes2Net	73.63%	79.29%	70.60%
Res2Net	72.83%	77.90%	68.40%
ResNetSe	74.15%	79.85%	70.94%
TDNN	74.89%	80.34%	71.63%
HTSAT	76.34%	82.82%	75.34%
Ours	**89.30%**	**94.74%**	**91.62%**

The best results are emphasized in bold.

**Figure 4:**
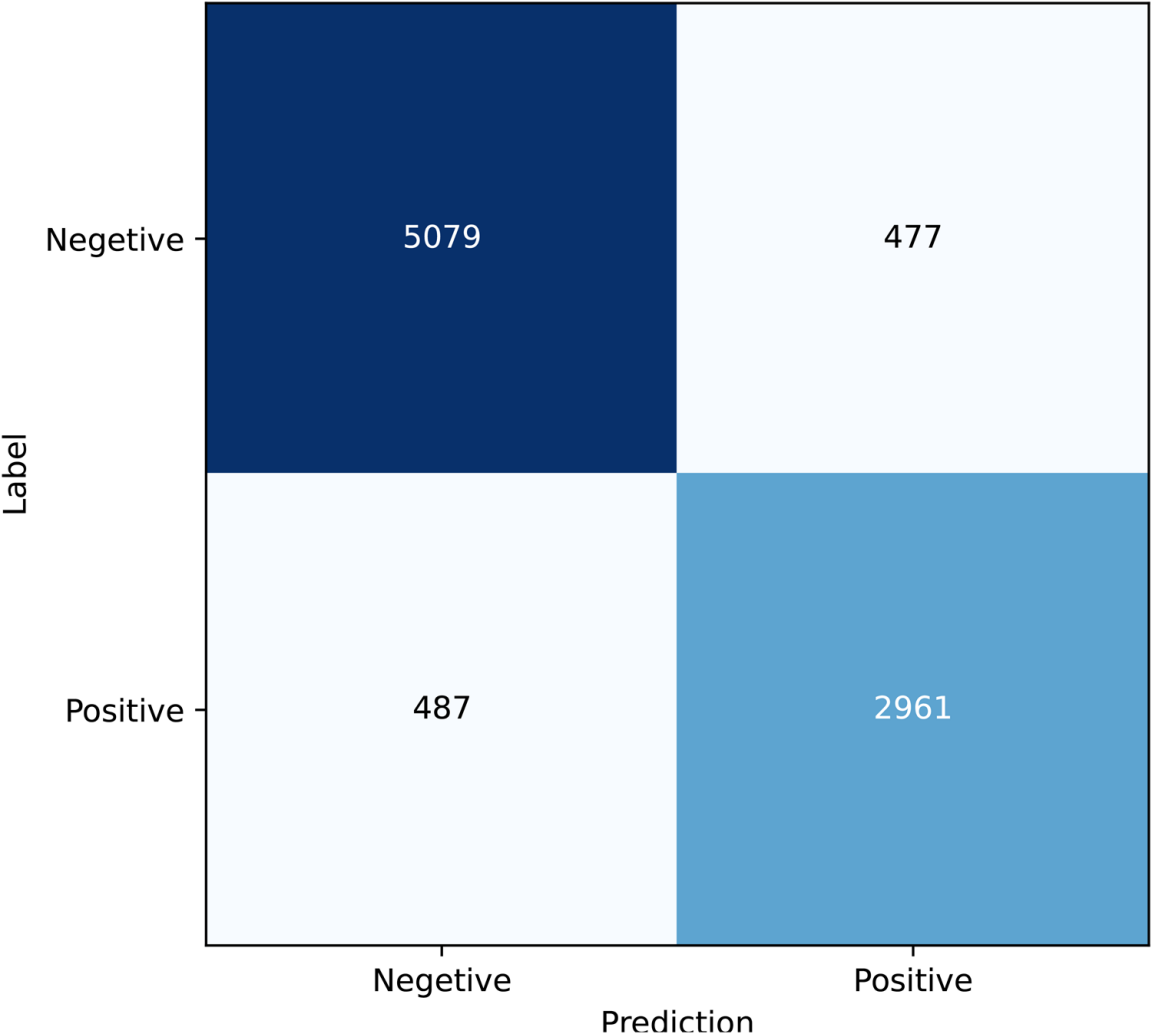
Confusion matrix.

A confusion matrix is a tool used to evaluate the performance of a classification model. It presents the relationship between the actual classes and the predicted classes in a tabular format, providing insights into the model’s performance for each category, including both correct and incorrect predictions. As shown in Figure 4, a significant number of samples are concentrated along the diagonal of the matrix, indicating that the majority of predictions are accurate.

### 4.5 Ablation experiment

In this section, the primary objectives of the ablation experiments are as follows: (1) To determine whether the improvement in detection performance originates from the symptom information or the attention mechanism. (2) To evaluate whether the network can predict symptom information to mitigate the impact of incorrect symptom inputs from patients. (3) To assess the effect of ablating different symptoms on the symptom attention mechanism. (4) To explore whether leveraging symptom pretraining, instead of symptom attention, can enhance the performance of COVID-19 detection.

#### 4.5.1 The effectiveness of clinical symptom prior information

**Figure 5:**
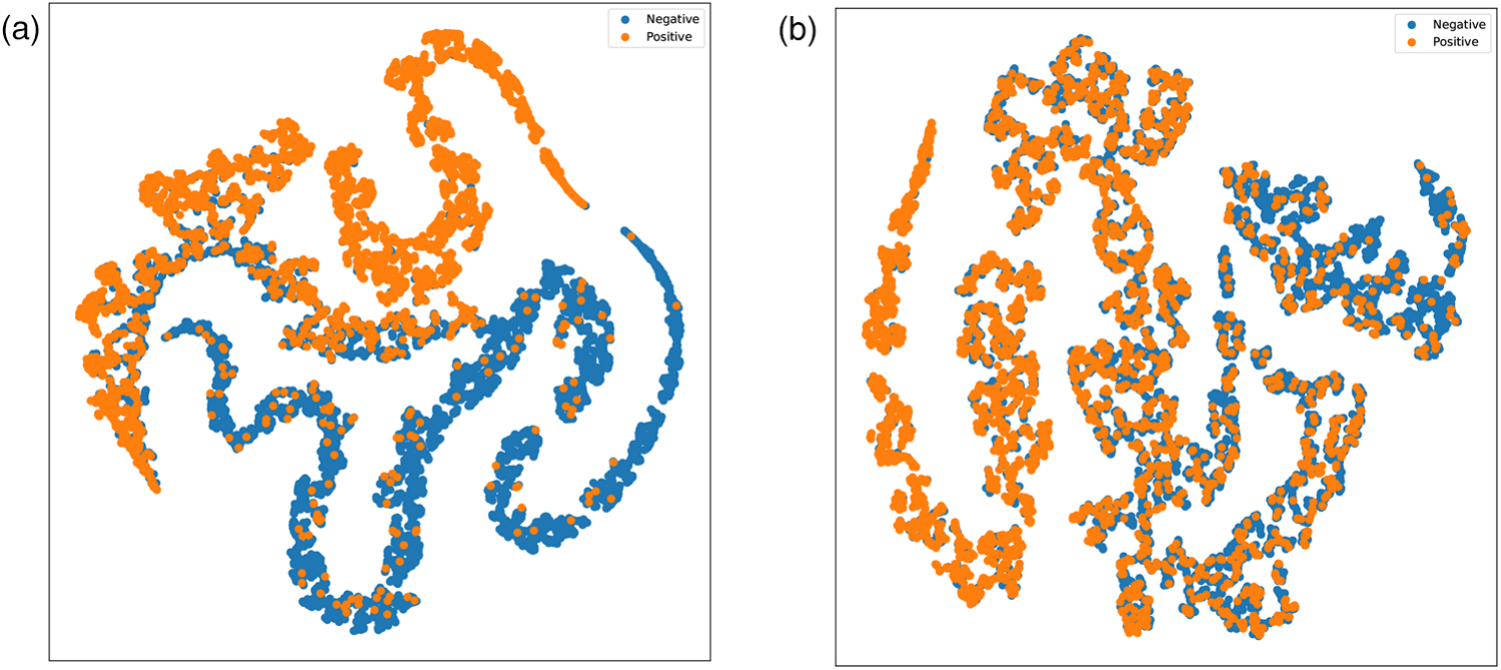
t-SNE visualizations of embeddings generated by **(a)** SympCoughNet and **(b)** SympCoughNet-ablated.

We designed two versions of ablation experiments to evaluate the impact of symptom attention on the model’s performance: one removes the entire symptom attention module, resulting in a model with pure audio input, referred to as SympCoughNet-ablated; the other uses randomly generated symptom information as input, referred to as SympCoughNet-random. As shown in Table 3, the SympCoughNet-ablated model, which relies solely on audio input, demonstrates competitive performance compared to the methods in Table 2, ranking second only to SympCoughNet in terms of AUROC. However, the SympCoughNet-random model, which introduces random symptom information, performs poorly despite having slightly more parameters than SympCoughNet-ablated. This result suggests that false or random symptom information acts as noise, negatively impacting the model’s ability to detect COVID-19. It highlights the critical importance of accurate and clinically meaningful symptom information in enhancing detection performance. We also present the t-SNE visualizations of SympCoughNet-ablated and SympCoughNet, as shown in Figure 5. The incorporation of symptom-based attention significantly enhances the quality of the embedding representation and mitigates domain shift. We trained a 3-layer MLP with layer dimensions of [64, 32, 2] using symptoms to predict whether a patient is positive, achieving an accuracy of 81.75%.

**Table 3:** The performance of the ablated symptom attention model and the random input symptom information model.

Method	ACC	AUROC	PR
SympCoughNet-random	69.91%	73.94%	64.11%
SympCoughNet-ablated	75.24%	81.27%	73.12%
SympCoughNet	89.30%	94.74%	91.62%

#### 4.5.2 The network can learn symptom biomarkers

**Figure 6:**
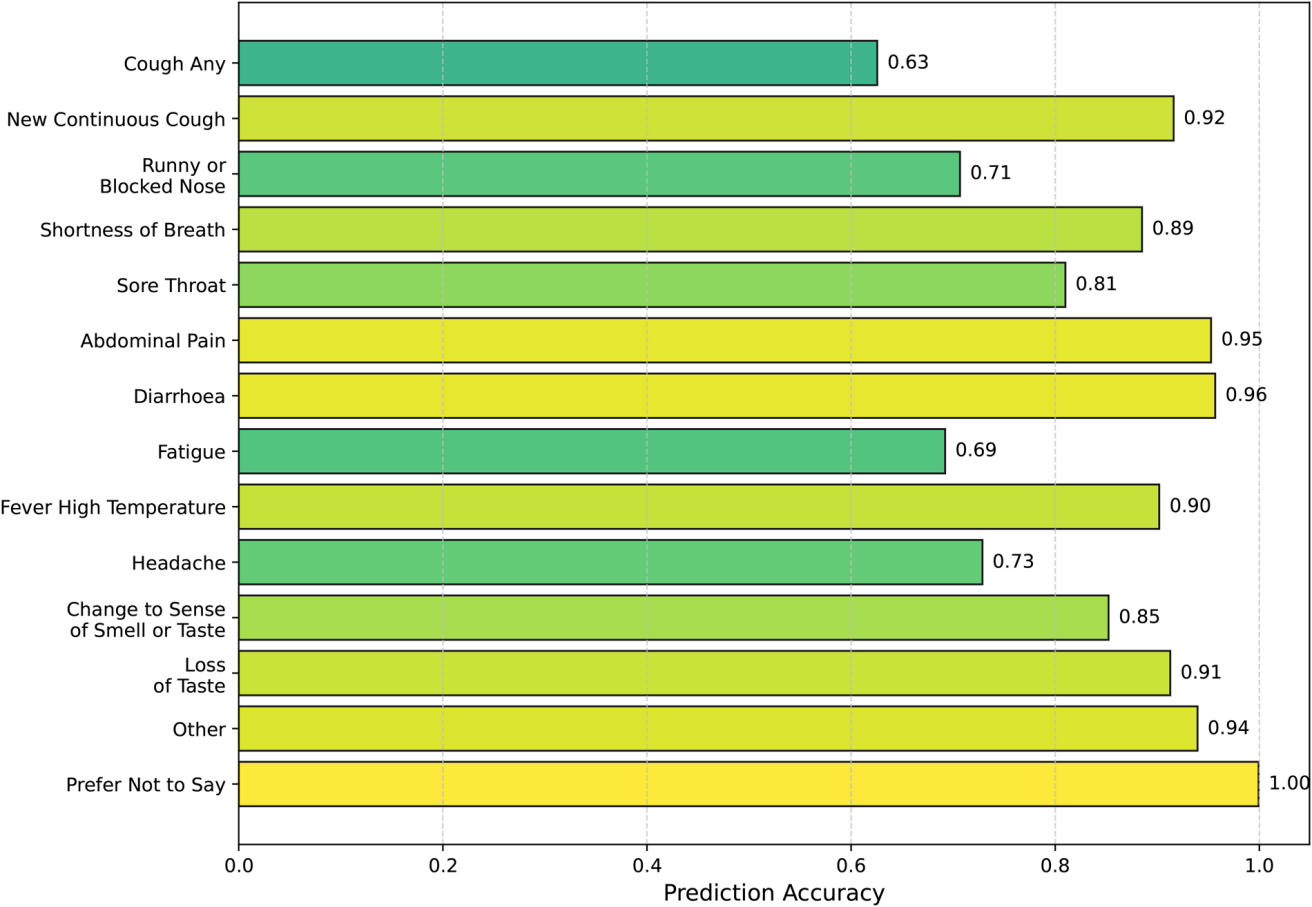
Symptom prediction accuracy.

Based on the findings, erroneous symptom information can potentially mislead the network and negatively affect its predictions. To address this issue, we leverage SympCoughNet-ablated, which relies solely on audio input, to directly predict symptom information in scenarios where patients may input incorrect or incomplete symptom prior knowledge. We denote this model as SympCoughNet-ablated-symp. As shown in Figure 6, the horizontal axis represents the prediction accuracy, while the vertical axis corresponds to the predicted symptom labels. The average prediction accuracy for all symptoms is 84.85%. Common COVID-19 indicators, such as “shortness of breath” and “fever high temperature,” are predicted with relatively high accuracy, achieving 81% and 90%, respectively. This demonstrates the model’s strong ability to infer critical symptoms directly from audio features. In contrast, symptoms like “fatigue” and “cough any” exhibit lower prediction accuracies, at 69% and 63%, respectively. Notably, “diarrhoea” and “abdominal pain” achieve the highest prediction accuracies, reaching 96% and 95%, respectively. These findings highlight the potential of audio-based symptom prediction as an effective supplementary approach to validate or correct patients’ provided symptom information.

The relatively lower prediction accuracy for “cough any” can be attributed to several factors. In multi-label classification tasks, there is often a trade-off between the prediction accuracies of different labels. During training, we used the average accuracy across all labels as the criterion for selecting the best model, which may have inadvertently prioritized overall performance over optimizing individual labels like “cough any.” Additionally, inferring cough symptoms directly from audio presents inherent challenges. The model may exhibit a bias towards assuming that most patients exhibit cough symptoms, potentially due to the limitations of cough audio features or biases in the training data. These factors highlight the challenges in achieving high accuracy for “cough any” and underscore the need for improved handling of label-specific trade-offs and more nuanced approaches to symptom-specific predictions. Nonetheless, these findings emphasize the potential of audio-based symptom prediction as an effective supplementary approach to validate or correct patients’ provided symptom information.

#### 4.5.3 Impact of different symptoms on COVID-19 detection performance

**Figure 7:**
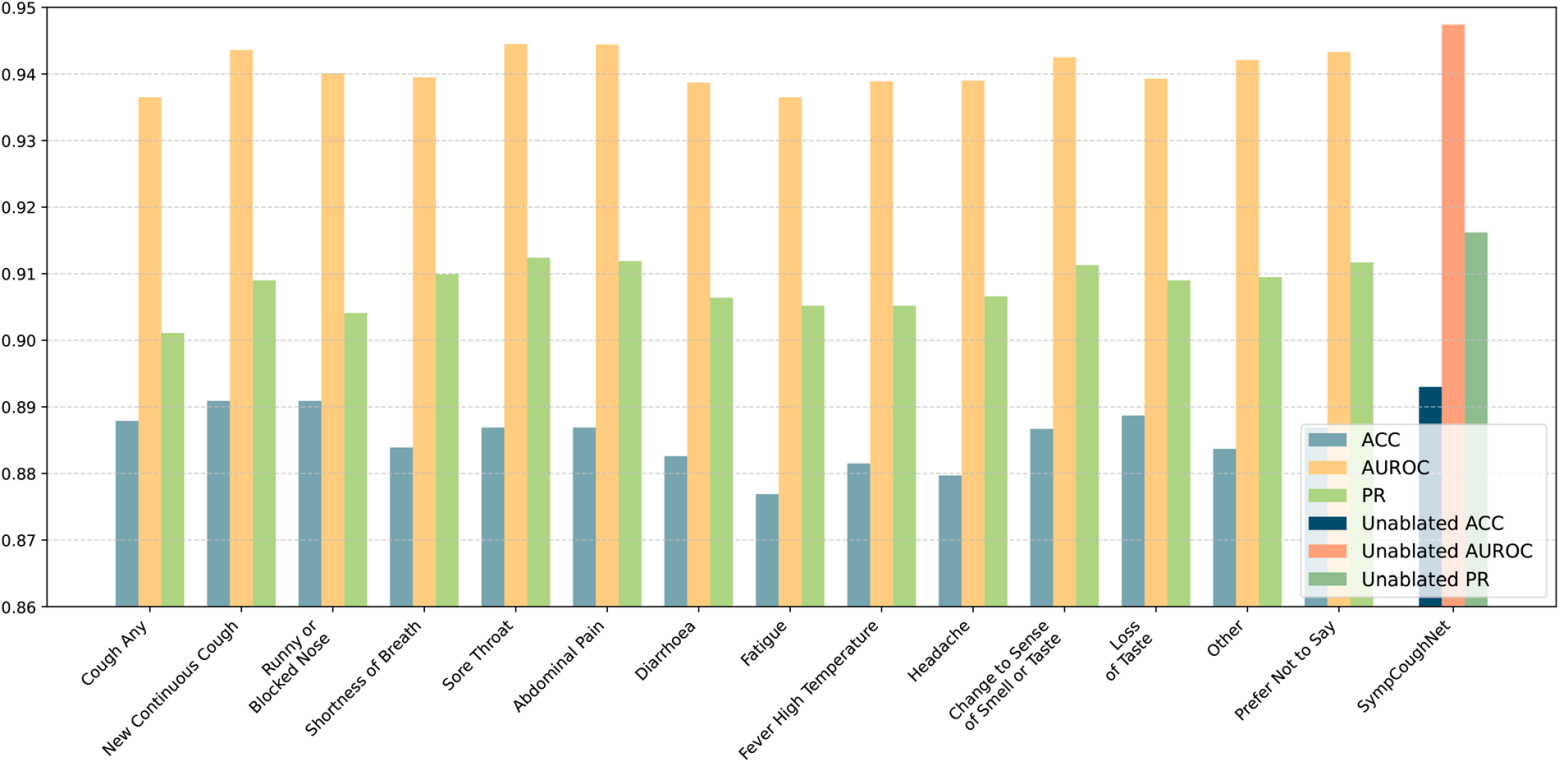
The performance of the model in predicting COVID-19 after ablating individual symptoms. The ablated symptoms, from left to right, are: “cough any,” “new continuous cough,” “runny or blocked nose,” “shortness of breath,” “sore throat,” “abdominal pain,” “diarrhoea,” “fatigue,” “fever high temperature,” “headache,” “change to sense of smell or taste,” “loss of taste,” “other,” and “prefer not to say.”

To verify which specific symptom is the most critical for detecting COVID-19, we conducted ablation experiments on each of the 14 symptoms and evaluated the model’s performance, as shown in Figure 7. While the overall performance does not appear to change significantly when a single symptom is removed, it is noteworthy that the decline in model performance seems to align with the model’s ability to predict the corresponding symptom. As presented in Table 4, ablating Fatigue and Headache results in the most significant impact on Accuracy and PR, particularly for Fatigue, where the ACC drops to 87.96%, the lowest value observed. At the same time, the SympCoughNet-ablated model performs poorly in predicting both Fatigue and Headache symptoms. Conversely, ablating Prefer Not to Say leads to the highest AUROC (94.36%), which aligns with the strong prediction performance of the SympCoughNet-ablated model for this symptom. This supports our hypothesis that CNNs can extract a limited amount of symptom information from audio. Building on this foundation, we apply symptom attention to amplify the weight of symptom-related features. Poor predictive performance for Fatigue and Headache suggests that the model struggles to extract information about these symptoms, leading to a more pronounced decline in performance when they are removed. Interestingly, the ablation of New Continuous Cough and Runny or Blocked Nose has the least impact on Accuracy. Simultaneously, the SympCoughNet-ablated model shows strong prediction performance for New Continuous Cough but weaker performance for Runny or Blocked Nose. This discrepancy might be due to trade-offs made during the model training process.

**Table 4:** The table presents accuracy (ACC), area under the receiver operating characteristic curve (AUROC), and precision-recall (PR) for models trained without specific symptoms.

Ablated symptoms	ACC	AUROC	PR
Cough any	88.79%	93.65%	90.11%
New continuous cough	89.09%	94.36%	90.90%
Runny or blocked nose	89.09%	94.01%	90.41%
Shortness of breath	88.36%	94.23%	90.99%
Sore throat	88.70%	94.45%	91.24%
Abdominal pain	88.69%	94.40%	91.19%
Diarrhoea	88.26%	93.87%	90.64%
Fatigue	87.96%	93.70%	90.15%
Fever high temperature	88.15%	93.89%	90.52%
Headache	87.97%	93.90%	90.66%
Change to sense of smell or taste	88.67%	94.25%	91.13%
Loss of taste	88.23%	93.93%	90.70%
Other	88.37%	94.21%	90.95%
Prefer not to say	88.69%	94.33%	91.17%

### 4.6 Symptom pretraining does not improve COVID-19 detection performance

In our approach to combining symptoms and audio, we also considered using the network SympCoughNet-ablated-symp, trained in Section 4.5.2, as a pre-trained model and fine-tuning it with only audio input. The idea was to investigate whether SympCoughNet-ablated-symp, which contains more symptom information after symptom pre-training, could enhance its ability to detect COVID-19. As shown in Table 5, we found that using symptoms as a pre-training approach did not provide any advantages. We also performed comparisons using contrastive learning (SupCon Loss) as a pre-training method. We found that neither Symptom Pre-training nor Contrastive Learning Pre-training significantly improved the performance of COVID-19 detection. This suggests that extracting symptom information through pre-training is quite challenging, and there may be forgetting of symptom information during the fine-tuning phase. However, by adopting a symptom attention mechanism to integrate symptom and audio information, we achieved a significant performance improvement. Although collecting symptom information requires additional effort, its contribution to improving detection performance is clearly evident.

**Table 5:** Symptom pre-training extracts limited symptom information (CL Pre-training refers to contrastive learning pre-training).

Pre-training method	ACC	AUROC	PR
Symptom pre-training	75.16%	80.62%	72.85%
CL pre-training	75.37%	81.05%	73.08%
No pre-training	7524%	81.27%	73.12%

## 5 Discussion

Recent studies have shown that audio-based classifiers for COVID-19 detection are highly influenced by confounding factors such as age, gender, and self-reported symptoms (12). These factors significantly impact model performance, and controlling for them often leads to reduced accuracy. Moreover, a smaller dataset size due to such controls may result in model underfitting. Since COVID-19 symptoms themselves serve as key discriminative features for distinguishing positive from negative cases, controlling for these factors during training may cause the model to inadvertently learn other confounding variables. Therefore, we controlled for factors like gender and self-reported symptoms during testing, with the results presented in Table 6. Note that we did not report results for the symptom category “prefer not to say” due to its insufficient representation in the dataset. Although we did not include age-related information during the training process, the experiments across different age groups and gender groups are still observable.

**Table 6:** Model performance under controlled testing conditions.

Controlled confounders	ACC	AUROC	PR
Gender			
Male	88.78%	94.21%	90.07%
Female	88.93%	95.19%	92.59%
Age			
18–44	86.79%	93.68%	93.95%
45–64	88.82%	94.71%	90.65%
65+	92.34%	93.81%	79.25%
Symptoms			
Cough any	84.14%	85.26%	94.24%
New continuous cough	90.44%	78.56%	96.63%
Runny or blocked nose	83.88%	84.30%	93.89%
Shortness of breath	86.89%	87.54%	95.37%
Sore throat	82.95%	80.90%	93.54%
Abdominal pain	89.46%	92.03%	97.44%
Diarrhoea	90.84%	88.42%	95.81%
Fatigue	86.58%	85.57%	95.35%
Fever high temperature	95.64%	72.20%	97.80%
Headache	87.44%	83.84%	95.70%
Change to sense of smell or taste	92.94%	78.06%	96.52%
Loss of taste	95.19%	63.71%	96.42%
Other	87.05%	80.85%	94.89%

## 6 Conclusion

In conclusion, SympCoughNet integrates symptom prior knowledge with audio features, providing a novel approach to enhance COVID-19 detection through cough audio analysis. By leveraging a symptom-encoded attention mechanism, the model effectively captures critical biological signals while mitigating the impact of irrelevant noise. This innovation addresses the low accuracy of traditional audio-based methods in detecting COVID-19 and the limitations of traditional symptom-based methods due to individual variability in symptomatic responses, thereby significantly improving detection performance, including accuracy, AUROC, and PR metrics. Our findings demonstrate the potential of combining symptom knowledge with audio-based detection, offering a cost-effective, rapid, and scalable solution for pandemic control, especially in resource-limited settings. Future work will explore the generalizability of SympCoughNet across other respiratory diseases and the integration of additional multimodal data to further improve robustness and applicability.

## Data Availability

The original contributions presented in the study are included in the article/Supplementary Material, further inquiries can be directed to the corresponding author.
